# Influence of cuff stiffness on hemodynamics and perceived cuff pressure in the upper extremities in males and females: implications for practical blood flow restriction training

**DOI:** 10.1186/s13102-023-00745-w

**Published:** 2023-10-19

**Authors:** Robert Bielitzki, Tom Behrendt, Toan Nguyen, Martin Behrens, Victoria Malczewski, Alexander Franz, Lutz Schega

**Affiliations:** 1 Department of Sport Science, Institute III, Otto-von-Guericke University Magdeburg, 39104 Magdeburg, Germany; 2 University of Applied Sciences for Sport and Management Potsdam, Olympischer Weg 7, 14471 Potsdam, Germany; 3 Department of Orthopedics and Trauma Surgery, University Hospital Bonn, 53127 Bonn, Germany

**Keywords:** Vascular occlusion, Elastic wrap, Blood flow velocity, Muscle oxygenation, Perceptual responses, NIRS

## Abstract

**Background:**

Practical blood flow restriction (pBFR) during exercise is a cost-saving alternative to traditional blood flow restriction using pneumatic cuffs, particularly when exercising in a group setting. Depending on the pBFR technique, several factors (e.g., cuff width, limb circumference) have already been shown to be of importance when applying the pBFR pressure. Given that elastic cuffs are often used for pBFR, the cuff stiffness might be an additional influencing factor. Therefore, the present study compared the acute effects of three elastic cuffs with identical width but different stiffness (high stiffness (HS), medium stiffness (MS), and low stiffness (LS)) on hemodynamic measures and perceived cuff pressure at rest.

**Methods:**

In a randomized, counter-balanced cross-over study, 36 young and normotensive participants completed three experimental trials. After a 10-min rest period in supine position, the cuff was loosely and proximally applied to the right upper arm. Following baseline data recording, the cuff was successively tightened in 10%-increments with respect to the limb circumference (%overlap) until arterial blood flow was occluded. At baseline and during each %overlap, systolic peak blood flow velocity of the brachial artery, rating of perceived cuff pressure, as well as muscle oxygen saturation and total hemoglobin concentration of the biceps brachii muscle were recorded.

**Results:**

The %overlap required to occlude arterial blood flow was different between the three cuffs (HS: 30.9 ± 3.8%, MS: 43.9 ± 6.1%, LS: 54.5 ± 8.3%). Furthermore, at 30% overlap, systolic peak blood flow velocity was lower when applying the HS (9.0 ± 10.9 cm∙s^− 1^) compared to MS (48.9 ± 21.9 cm∙s^− 1^) and LS cuff (62.9 ± 19.1 cm∙s^− 1^). Rating of perceived cuff pressure at 30% overlap was higher when using the HS (6.5 ± 1.5 arbitrary unit (a.u.)) compared to MS (5.1 ± 1.4 a.u.) and LS cuff (4.9 ± 1.5 a.u.) with no difference between the MS and LS cuff. However, muscle oxygen saturation and total hemoglobin concentration were not different between the three cuffs.

**Conclusions:**

The present study revealed that the cuff stiffness influenced blood flow velocity and arterial occlusion pressure. Therefore, cuff stiffness seems an important factor for the application of pBFR.

## Introduction

During the last decade, practical blood flow restriction (pBFR) training has emerged as an alternative to traditional blood flow restriction (BFR) using pneumatic cuffs to promote muscular adaptations (e.g., muscle strength and thickness [1–3]), since the costs for required equipment are lower and it can be easily applied in group settings [4]. During pBFR training, a non-pneumatic cuff (i.e., elastic [5] or rigid cuff [6]) is applied to the proximal part of the limb to generate a modest reduction in arterial inflow and a strong reduction up to full occlusion in venous return of the blood [7]. Among other mechanisms, venous blood pooling is thought to induce local hypoxia that can increase the exercise stimulus and training effect [8, 9]. Thus, the cuff pressure is thought to be of importance to induce the local hypoxic milieu and, in turn, beneficial adaptations without increasing the risk of adverse events [9, 10].

In order to produce an effective restriction pressure with a non-pneumatic cuff, different pBFR techniques have been developed such as the absolute (i.e., pressure is set based on a fixed overlap value) and relative overlap technique (i.e., pressure is set based on the overlap in relation to the limb circumference), or the perceived pressure technique (i.e., pressure is set based on participant’s pressure perception) [4]. When using the relative overlap technique [11, 12], an elastic cuff is tightened to a certain percentage overlap of the limb circumference to induce an individually tailored pBFR stimulus. Due to the application of the cuff with respect to limb circumference, this procedure is individualized and well reproducible, but also depends on the cuffs’ characteristics (e.g., cuff width) [4]. For instance, it was shown that wider cuffs require less pressure to occlude arterial blood flow compared to narrower cuffs [13, 14]. However, it still remains unclear if other cuff characteristics such as mechanical material properties (e.g., cuff stiffness, which can be defined as the slope of the curve in the elastic region [15]) may also influence the amount of BFR. Loenneke et al. [16] did not found differences in repetitions to failure or rate of perceived exertion between an elastic and a rigid nylon cuff with the same cuff width when performing three sets of knee extensions at 30% of individuals’ one repetition maximum (1RM). Furthermore, Buckner et al. [17] compared the influence of different materials of pneumatic cuffs on changes in isometric maximal voluntary contraction strength and muscle activity after four sets of unilateral biceps curls until failure at 30% of individuals’ 1RM. The authors found that cuffs of different material (i.e., narrow nylon (5 cm) versus elastic cuff (3 cm)), but comparable width, induced similar acute motor performance fatigue and physiological changes when a pressure of 40% of resting arterial occlusion pressure (AOP) was applied. However, to date no study has investigated the influence of different materials of non-pneumatic elastic cuffs typically used for pBFR.

Therefore, the aim of this study was to compare the influence of three non-pneumatic elastic cuffs with different stiffness (high stiffness (HS), medium stiffness (MS), low stiffness (LS)) on the percentage overlap (%overlap) to arterial occlusion (OTO), systolic peak blood flow velocity (v_sys_), muscle oxygenation, and perceived cuff pressure in the upper extremities in young males and females. We hypothesized that cuff stiffness would affect the hemodynamic and perceptual responses, i.e., a lower OTO, v_sys_, and muscle oxygenation, but a higher perceived cuff pressure at the same %overlap when applying the HS cuff compared to the cuffs with less stiffness. Considering that females may have a higher muscle oxygenation due to a greater vasodilatory response and greater proportional area of type I muscle fibers [18] as well as a higher pain sensitivity and lower pressure pain threshold [19] compared to males, we assumed sex differences in muscle oxygenation and perceived cuff pressure.

## Methods

### Subjects

As there was no comparable study with available effect sizes, we assumed a medium effect size (f = 0.25) for the changes in v_sys_. Based on a sample size calculation (using G*Power 3.1) for a repeated measures analysis of variance (ANOVA) (cuff (3) × overlap (4)) with α = 0.05, 1-β = 0.8, and correlation among repeated measurements = 0.4 a total of 36 young, healthy males (N = 18) and females (N = 18) were recruited for the present study, which represents a similar sample size to a comparable experiment by Mouser et al. [13]. All participants were free from (i) hypertension (< 140/90 mmHg), (ii) neurological, mental, and cardiovascular disorders or diseases, (iii) medication with central nervous or cardiovascular effects, (iv) open wounds or sensitive scar tissue at the right upper limb, and (v) had a skinfold thickness above the biceps brachii muscle of < 25 mm. All participants have given their written informed consent for participation. The study was approved by the local Ethics Committee (152/22) and conformed with the principles of the Declaration of Helsinki on human experimentation.

### Experimental design

**Fig. 1:**
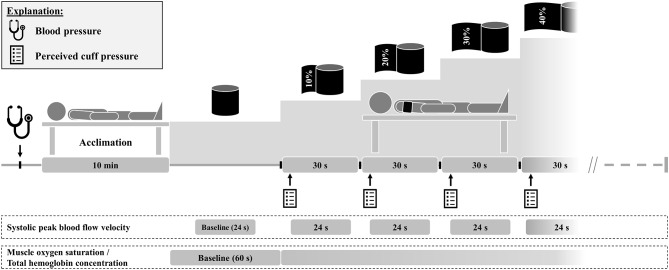
Schematic overview of the experimental procedure

In a randomized, counterbalanced cross-over design, participants completed three laboratory visits (separated by at least 24 h) during which a standardized occlusion protocol was applied using three elastic cuffs with different amounts of stiffness (i.e., HS, MS, and LS) but identical width (5 cm) and length (64 cm). At the first visit, participants’ anthropometric data were collected and they were familiarized with the experimental procedure. Upon arrival at each testing session, participants’ blood pressure was measured in seated position. Subsequently, participants were placed in a supine position with the right arm slightly abducted resting on a foam pad. During a 10-min rest period, participants were comprehensively familiarized with the perceived pressure scale. Afterwards, participants’ resting v_sys_ and muscle oxygenation data were collected. Thereafter, the HS cuff (MS and LS cuff layered on top of each other), MS cuff (BFR Bands, MuscleForge, Krakow, Malopolska, Poland), or LS cuff (Blood Flow Restriction Bands, Armageddon Sports, Dover, DE, USA) was loosely applied to the proximal part of the right upper arm. Comparable to Mouser et al. [13], the cuff was tightened by 10% of the upper arm circumference (i.e., 10% overlap), maintained for 30 s, and then tightened by further 10% (i.e., 20% overlap). This procedure was continued until the arterial blood flow could no longer be detected. During each %overlap, v_sys_, muscle oxygenation, and perceived cuff pressure were assessed (Fig. 1). All test sessions were conducted at the same time of day to minimize the effects of circadian variations. Participants were instructed to avoid consuming alcohol or pain medication as well as strenuous exercise 24 h before each trial. Furthermore, participants were briefed to have their last meal and caffeine intake at least 2 and 8 h before the experimental sessions, respectively. Ambient temperature and relative humidity were kept constant during the experiments.

### Cuff stiffness

**Fig. 2:**
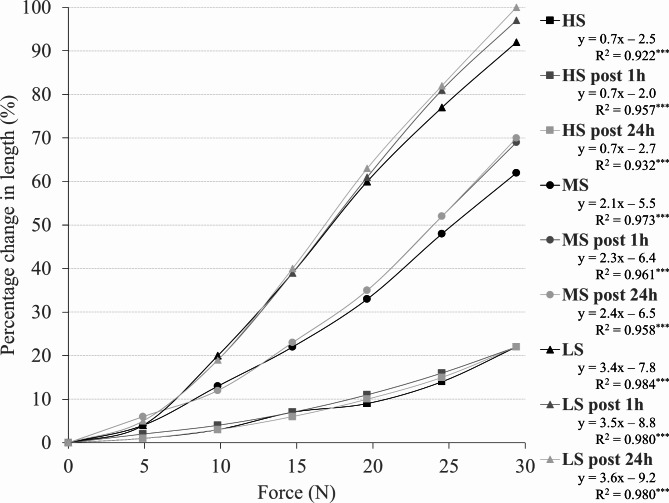
Force-elongation relationship with weight (x-axis) and percentage change in length (y-axis) to determine the stiffness of the high stiffness (HS), medium stiffness (MS), and low stiffness (LS) cuff. The equation of the linear regression curve (y = ax – b) with the corresponding regression coefficient (R^2^) and level of significance (p) are presented for each curve. Significant regression is presented as ^***^p < 0.001

Similar to Abe et al. [11], a force-elongation curve was plotted to quantify the cuff stiffness. For that purpose, one end of each cuff was attached to a wall and the other was fitted with a clamp. Subsequently, a 10-cm distance was marked on the cuffs. A weight of 0.5 kg was applied to the clamp and increased stepwise by 0.5 kg. After each additional weight applied, the previously marked distance was measured and the change in length was recorded. This procedure was performed until the force-elongation curve of the cuff with the lowest stiffness (i.e., LS) was no longer linear (i.e., until 3.0 kg). According to Abe et al. [11], a linear function was constructed with the x-axis representing the applied weight and the y-axis showing the percentage change in length. Additionally, the procedure was repeated 1 and 24 h after the initial testing to check for reliability of the stiffness measurements. A linear force-elongation relationship was found for all three cuffs (Fig. 2) with an excellent reliability between the three trials (HS cuff: intraclass correlation coefficient (ICC)_3,1_ = 0.996, MS cuff: ICC_3,1_ = 0.996, LS cuff: ICC_3,1_ = 0.998).

### Systolic peak blood flow velocity

During the experimental sessions, v_sys_ was measured using a handheld, bidirectional, and highly sensitive 8 MHz Doppler probe (Dopplex DMX, Huntleigh Healthcare Ltd, Cardiff, UK). The probe was placed over the brachial artery in the antecubital fossa with an insonation angle of 45–60° opposite to the direction of flow according to the manufacturer’s manual. Measurements were performed at baseline (0% overlap) and during each %overlap for 24 s. Intersession reliability for the baseline measurements was good (ICC_3,k_ = 0.843).

### Rating of perceived cuff pressure

Rating of perceived cuff pressure (RPP) was assessed using the perceived pressure scale [5], since it is a common tool used for the application of elastic cuffs for pBFR. Participants were asked to rate the intensity of the pressure induced by the elastic cuffs. Prior to the testing procedure, participants were given written instructions including specific descriptions and anchors (i.e., 0/10: no pressure at all, 3/10: light pressure, 7/10: moderate pressure without pain, 10/10: intensive pressure with clearly noticeable pain [20]). Participants were also briefed to accurately differentiate between pain sensations distal to the cuff (e.g., ischemia-induced tissue pain) and the mechanical pressure induced by the cuffs. RPP (expressed in arbitrary unit (a.u.)) was queried at the beginning of each %overlap.

### Muscle oxygenation

Total hemoglobin concentration (tHb) and oxygenated hemoglobin as percentage of tHb (muscle oxygen saturation, S_m_O_2_) were recorded using a muscular near-infrared spectroscopy (mNIRS) device (MOXY, Fortiori Design LLC, Hutchinson, USA). Initially, the respective area was cleaned with disinfectant. Afterwards, the mNIRS monitor (61 × 44 × 21 mm, 48 g) was placed on the muscle belly of the biceps brachii and fixed with elastic adhesive tape to guarantee a similar contact pressure between the trials. The position of the monitor was marked for the subsequent sessions. S_m_O_2_ and tHb were recorded for 60 s at baseline (0% overlap) and throughout the test protocol. The mNIRS data were recorded with a sampling rate of 2 Hz. Data were averaged for baseline recordings and across each %overlap. Intersession reliability of S_m_O_2_ (ICC_3,k_ = 0.828) and tHb (ICC_3,k_ = 0.976) recorded at baseline was good and excellent, respectively.

### Statistical analysis

Statistical analyses were conducted using JASP Statistics (Version 0.16.4, University of Amsterdam, Amsterdam, Netherlands). All data were screened for normality of distribution and homogeneity of variance using the Shapiro-Wilk and Levene´s tests, respectively. Because studies have shown that repeated measures ANOVAs [21] are robust to moderate violation of normality and homogeneity, nonparametric tests were not used to test for differences. Subsequently, three-way repeated measures ANOVAs (overlap × cuff × sex) were conducted for v_sys_, RPP, S_m_O_2_, and tHb, including baseline (0% overlap) and the first three %overlaps (10% overlap, 20% overlap, 30% overlap). This approach was used, because arterial blood flow of the most participants was already occluded at 40% overlap when the HS cuff was applied. Furthermore, a two-way ANOVA with repeated measures (cuff × sex) was performed for OTO. The effect size partial eta squared (*η*_*p*_^2^) was calculated and interpreted as follows: small ≥ 0.01, medium ≥ 0.06, and large effect size ≥ 0.14 [22]. Greenhouse-Geisser correction was applied in case of sphericity violation. In case of significant interaction or main effects, post-hoc tests with Bonferroni correction were performed. Furthermore, the effect size Cohens’ d (*d*) was computed and interpreted according to Cohen [22]: small ≥ 0.20, medium ≥ 0.50, and large effect size ≥ 0.80. Mean differences (MD) and 95% confidence intervals (95% CI) were provided and the level of significance was set at *p* < 0.05.

## Results

All participants (demographic and anthropometric data are presented in Table 1) successfully completed the experimental sessions without adverse events, except some cases of slight tingling in the fingers at the end of the measurements. Regarding OTO, three males had to be excluded from data analyses because the occlusion of arterial blood flow was not possible with the MS and/ or LS cuff due to painful skinfold pinching and cuff stretch up to the yield point. Furthermore, one female was excluded from data analyses for v_sys_, RPP, S_m_O_2_, and tHb because arterial blood flow was already occluded at 20% overlap using the HS cuff.

**Table 1:** Participants’ characteristics expressed as means ± standard deviations

		*N = 36 (18 males / 18 females)*
**Age (yrs)**		21.7 ± 2.3
**Weight (kg)**		73.3 ± 14.9
**Height (cm)**		173.1 ± 10.1
**Body mass index (kg ∙ m** ^**− 2**^ **)**		24.3 ± 3.1
**Systolic blood pressure (mmHg)**	**High stiffness cuff**	119.9 ± 8.9
	**Medium stiffness cuff**	122.9 ± 7.6
	**Low stiffness cuff**	123.6 ± 7.1
**Diastolic blood pressure (mmHg)**	**High stiffness cuff**	76.9 ± 6.4
	**Medium stiffness cuff**	78.9 ± 5.2
	**Low stiffness cuff**	78.6 ± 4.7

### Overlap to occlusion

**Fig. 3:**
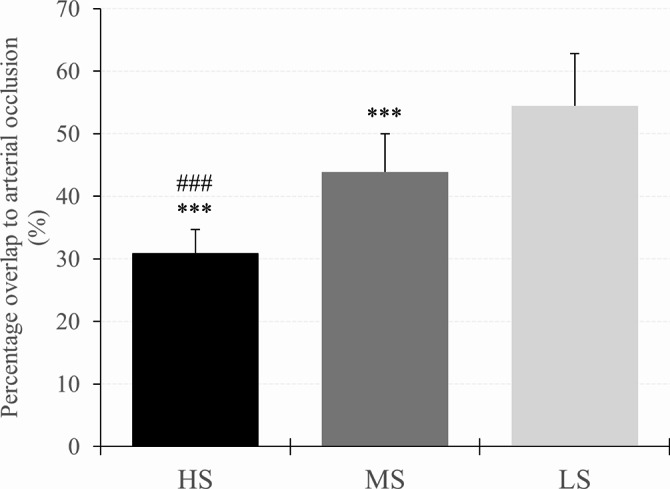
Percentage overlap needed for arterial occlusion in the cuff with high stiffness (HS), medium stiffness (MS), and low stiffness (LS). Significant difference to LS and MS is presented as ^*^p < 0.05, ^**^p < 0.01, ^***^p < 0.001 and ^#^p < 0.05, ^##^p < 0.01, ^###^p < 0.001, respectively

There was a main effect of cuff (*F*_*2,62*_ = 175.679, *p* < 0.001, *η*_*p*_^2^ = 0.850) and post-hoc analysis indicated that OTO was lower with the HS compared to the MS (MD = -13.06% (-16.18 to -9.93%), *p* < 0.001, *d* = 2.06) and LS cuff (MD = -23.78% (-26.90 to -20.65%), *p* < 0.001, *d* = 3.75). Moreover, OTO was also lower when using the MS compared to the LS cuff (MD = -10.72% (-13.85 to -7.60%), *p* < 0.001, *d* = 1.69). Descriptive data are shown in Table 2; Fig. 3.

**Table 2:** Hemodynamic, physiological, and perceptual responses to progressive practical blood flow restriction pressures (10%, 20%, and 30% overlap in relation to the individuals’ upper arm circumference) using a cuff with a high stiffness (HS), medium stiffness (MS), and low stiffness (LS). Data are expressed as means ± standard deviations

		HS	MS	LS
**Relative overlap to arterial occlusion (%)**		30.86 ± 3.74	44.00 ± 6.04	54.57 ± 8.17
**Systolic peak blood flow velocity (cm · s** ^**− 1**^ **)**	**Baseline (0% overlap)**	71.58 ± 14.63	70.26 ± 15.98	73.86 ± 13.69
	**10% overlap**	71.39 ± 14.08	68.39 ± 15.24	70.43 ± 11.24
	**20% overlap**	51.18 ± 20.10^†††^	67.92 ± 13.05	68.65 ± 15.44
	**30% overlap**	8.99 ± 10.59^†††^	48.86 ± 21.86^†††^	62.88 ± 19.06^†††^
**Rate of perceived cuff pressure** **(arbitrary unit)**	**Baseline (0% overlap)**	0.09 ± 0.28	0.03 ± 0.17	0.00 ± 0.00
	**10% overlap**	1.66 ± 0.97^†††^	1.57 ± 0.95^†††^	1.46 ± 0.74^†††^
	**20% overlap**	3.89 ± 1.28^†††^	3.34 ± 1.08^†††^	3.17 ± 1.18^†††^
	**30% overlap**	6.49 ± 1.50^†††^	5.06 ± 1.39^†††^	4.94 ± 1.47^†††^
**Muscle oxygen saturation (%)**	**Baseline (0% overlap)**	66.90 ± 8.35	66.32 ± 7.33	67.28 ± 7.12
	**10% overlap**	64.53 ± 9.90	64.24 ± 8.56	65.82 ± 7.22
	**20% overlap**	56.97 ± 11.40^†††^	58.03 ± 9.36^†††^	59.81 ± 7.81^†††^
	**30% overlap**	49.46 ± 11.73^†††^	51.41 ± 9.13^†††^	53.50 ± 7.99^†††^
**Total hemoglobin concentration** **(arbitrary unit)**	**Baseline (0% overlap)**	12.61 ± 0.36	12.62 ± 0.37	12.65 ± 0.32
	**10% overlap**	12.66 ± 0.39^†^	12.66 ± 0.39	12.66 ± 0.35
	**20% overlap**	12.75 ± 0.42^†††^	12.72 ± 0.43^†††^	12.72 ± 0.39^†††^
	**30% overlap**	12.84 ± 0.43^†††^	12.79 ± 0.44^†††^	12.80 ± 0.40^†††^

Significant difference to baseline: ^†^p < 0.05, ^††^p < 0.01, ^†††^p < 0.001

### Systolic peak blood flow velocity

**Fig. 4:**
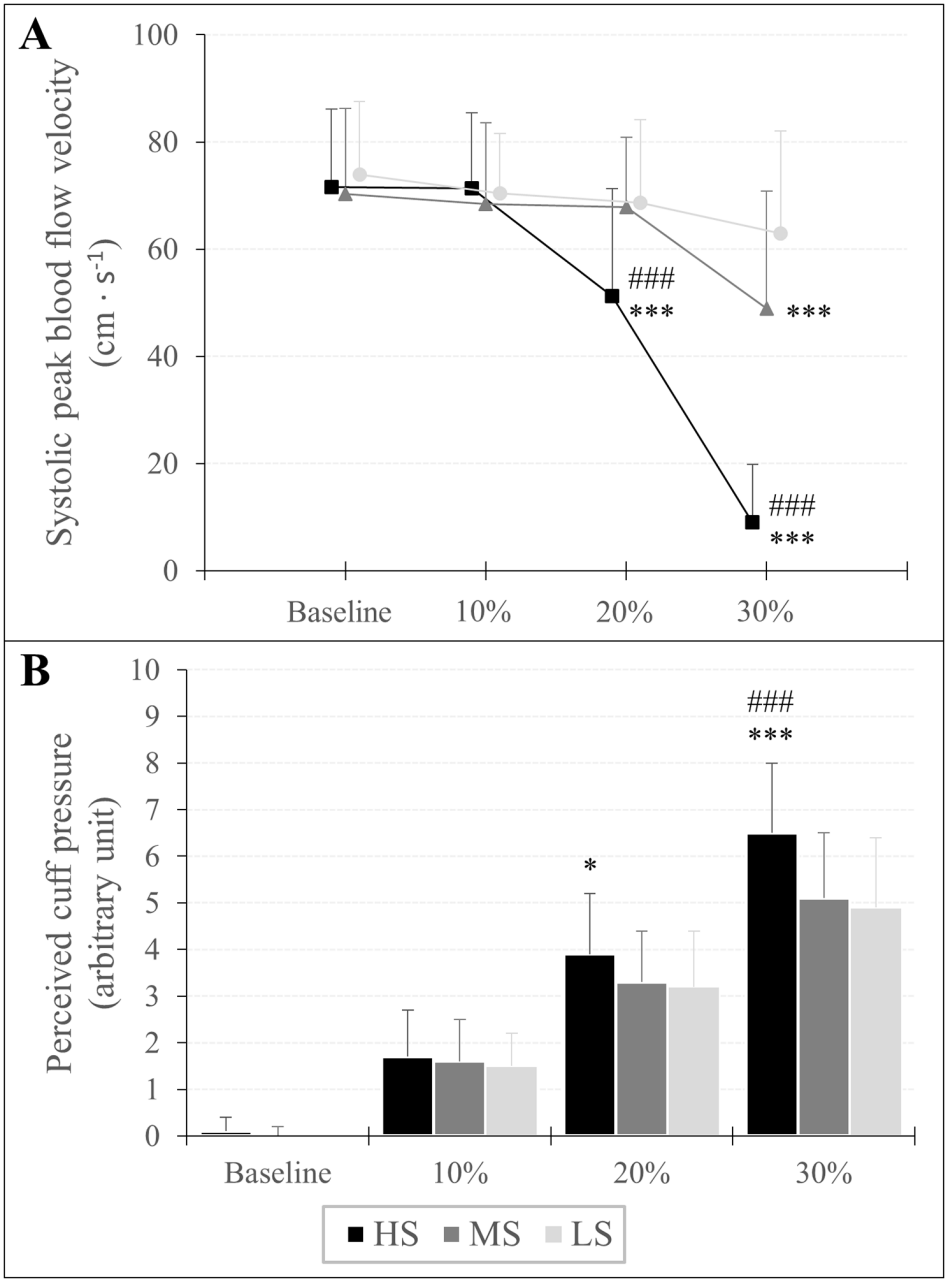
Systolic peak blood flow velocity **(A)** and rating of perceived cuff pressure **(B)** in response to progressive practical blood flow restriction pressures expressed as percentage overlap in relation to the individuals’ arm circumference. Significant difference to LS and MS is presented as ^*^p < 0.05, ^**^p < 0.01, ^***^p < 0.001 and ^#^p < 0.05, ^##^p < 0.01, ^###^p < 0.001, respectively

There was an overlap × cuff interaction (*F*_*3.642,120.192*_ = 71.952, *p* < 0.001, *η*_*p*_^*2*^ = 0.686) as well as a main effect of overlap (*F*_*1.770,58.422*_ = 161.427, *p* < 0.001, *η*_*p*_^*2*^ = 0.830) and cuff (*F*_*2,66*_ = 50.380, *p* < 0.001, *η*_*p*_^*2*^ = 0.604) for v_sys_. Post-hoc analysis revealed that v_sys_ was lower at 30% overlap in each cuff compared to baseline (HS: MD = -62.67 cm · s^− 1^ (-70.97 to -54.37 cm · s^− 1^), *p* < 0.001, *d* = 3.95; MS: MD = -21.53 cm · s^− 1^ (-29.83 to -13.24 cm · s^− 1^), *p* < 0.001, *d* = 1.36; LS: MD = -11.02 cm · s^− 1^ (-19.31 to -2.72 cm · s^− 1^), *p* < 0.001, *d* = 0.69). Furthermore, when the HS cuff was applied, v_sys_ was also lower at 20% overlap compared to baseline (MD = -20.51 cm · s^− 1^ (-28.80 to -12.21 cm · s^− 1^), *p* < 0.001, *d* = 1.29). Regarding cuff differences, v_sys_ was lower at 20% and 30% overlap when the HS cuff was used compared to the MS (MD = -16.89 cm · s^− 1^ (-26.08 to -7.70 cm · s^− 1^), *p* < 0.001, *d* = 1.07 and MD = -39.91 cm · s^− 1^ (-49.11 to -30.71 cm · s^− 1^), *p* < 0.001, *d* = 2.52, respectively) and the LS cuff (MD = -17.60 cm · s^− 1^ (-26.80 to -8.41 cm · s^− 1^), *p* < 0.001, *d* = 1.11 and MD = -53.99 cm · s^− 1^ (-63.18 to -44.79 cm · s^− 1^), *p* < 0.001, *d* = 3.40, respectively). Furthermore, v_sys_ was also lower at 30% overlap using the MS compared to the LS cuff (MD = -14.08 cm · s^− 1^ (-23.27 to -4.88 cm · s^− 1^), *p* < 0.001, *d* = 0.89). Descriptive data are shown in Table 2; Fig. 4.

### Rating of perceived cuff pressure

An overlap × cuff interaction (*F*_*3.946,130.209*_ = 13.994, *p* < 0.001, *η*_*p*_^*2*^ = 0.298) as well as a main effect of overlap (*F*_*1.668,55.046*_ = 674.771, *p* < 0.001, *η*_*p*_^*2*^ = 0.953) and cuff (*F*_*2,66*_ = 11.067, *p* < 0.001, *η*_*p*_^*2*^ = 0.251) was found for RPP. Post-hoc analysis showed that RPP increased in each %overlap stage compared to baseline for all three cuffs (HS10%: MD = 1.57 a.u. (0.98 to 2.17 a.u.), *p* < 0.001, *d* = 1.51; HS20%: MD = 3.80 a.u. (3.21 to 4.40 a.u.), *p* < 0.001, *d* = 3.66; HS30%: MD = 6.41 a.u. (5.82 to 7.00 a.u.), *p* < 0.001, *d* = 6.16; MS10%: MD = 1.54 a.u. (0.94 to 2.13 a.u.), *p* < 0.001, *d* = 1.48; MS20%: MD = 3.32 a.u. (2.72 to 3.91 a.u.), *p* < 0.001, *d* = 3.19; MS30%: MD = 5.03 a.u. (4.44 to 5.63 a.u.), *p* < 0.001, *d* = 4.84; LS10%: MD = 1.46 a.u. (0.87 to 2.05 a.u.), *p* < 0.001, *d* = 1.40; LS20%: MD = 3.18 a.u. (2.59 to 3.77 a.u.), *p* < 0.001, *d* = 3.05; LS30%: MD = 4.95 a.u. (4.36 to 5.55 a.u.), *p* < 0.001, *d* = 4.76). Regarding differences between cuffs, RPP was higher using the HS cuff with 20% overlap compared to the LS cuff (MD = 0.71 a.u. (0.06 to 1.37 a.u.), *p* = 0.016, *d* = 0.69) as well as at 30% overlap compared to the MS (MD = 1.43 (0.78 to 2.09 a.u.), *p* < 0.001, *d* = 1.38) and LS cuff (MD = 1.54 a.u. (0.89 to 2.19 a.u.), *p* < 0,001, *d* = 1.48). Descriptive data are presented in Table 2; Fig. 4.

### Muscle oxygenation

*S*_*m*_*O*_*2*_: There was an overlap × cuff interaction (*F*_*2.374,78.326*_ = 3.232, *p* = 0.037, *η*_*p*_^*2*^ = 0.089) as well as a main effect of overlap (*F*_*1.297,42.808*_ = 404.914, = *p* < 0.001, *η*_*p*_^*2*^ = 0.925) and sex (*F*_*1,33*_ = 5.096, *p* = 0.031, *η*_*p*_^*2*^ = 0.134) for S_m_O_2_. Post-hoc analysis revealed that S_m_O_2_ was lower at 20% overlap (HS: MD = -9.94% (-12.43 to -7.45%), *p* < 0.001, *d* = 1.15; MS = -8.28% (-10.77 to -5.78%), *p* < 0.001, *d* = 0.96; LS: MD = -7.42% (-9.98 to -5.00%), *p* < 0.001, *d* = 0.87) and 30% overlap (HS: MD = -17.46% (-19.95 to -14.97%), *p* < 0.001, *d* = 2.02; MS: MD = -14.91% (-17.42 to -12.42%), *p* < 0.001, *d* = 1.72; LS: MD = -13.79% (-16.28 to -11.30%), *p* < 0.001, *d* = 1.59) compared to baseline. Furthermore, the main effect of sex indicated that, regardless of the overlap and used cuff, S_m_O_2_ was lower in males compared to females (MD = -5.16% (-9.80 to -0.51%), *p* = 0.031, *d* = 0.60).

*tHb*: An overlap × cuff (*F*_*3.072,101.386*_ = 6.440, *p* < 0.001, *η*_*p*_^*2*^ = 0.163) and overlap × sex interaction (F_1.187,39.158_ = 14.814, *p* < 0.001, *η*_*p*_^*2*^ = 0.310) as well as a main effect of overlap (*F*_*1.187,39.158*_ = 117.125, = *p* < 0.001, *η*_*p*_^*2*^ = 0.780) and sex (*F*_*1,33*_ = 27.981, *p* < 0.001, *η*_*p*_^*2*^ = 0.459) was found for tHb. Post-hoc tests showed that tHb was higher at 20% overlap (HS: MD = 0.13 a.u. (0.09 to 0.18 a.u.), *p* < 0.001, *d* = 0.45; MS: MD = 0.10 a.u. (0.05 to 0.14 a.u.), *p* < 0.001, *d* = 0.32; LS: MD = 0.06 a.u. (0.02 to 0.11 a.u.), *p* < 0.001, *d* = 0.20) and 30% overlap (HS: MD = 0.22 a.u. (0.18 to 0.27 a.u.), *p* < 0.001, *d* = 0.75; MS: MD = 0.17 a.u. (0.12 to 0.21 a.u.), *p* < 0.001, *d* = 0.55; LS: MD = 0.15 a.u. (0.10 to 0.19 a.u.), *p* < 0.001, *d* = 0.45) compared to baseline. Moreover, tHb was higher already at 10% overlap using the HS (MD = 0.05 a.u. (0.00 to 0.09 a.u.), *p* = 0.018, *d* = 0.16) compared to baseline. Regarding sex differences, post-hoc analysis revealed that, regardless of the cuff, tHb was higher at 10% (MD = 0.06 a.u. (0.01 to 0.10 a.u.), *p* = 0.003, *d* = 0.20), 20% (MD = 0.15 a.u. (0.11 to 0.20 a.u.), *p* < 0.001, *d* = 0.52), and 30% overlap (MD = 0.24 a.u. (0.19 to 0.29 a.u.), *p* < 0.001, *d* = 0.80) in males, while in females tHb was higher only during 30% overlap compared to baseline (MD = 0.12 a.u. (0.07 to 0.16 a.u.), *p* < 0.001, *d* = 0.39). Furthermore, tHb was higher in males compared to females during baseline (MD = 0.51 a.u. (0.31 to 0.70 a.u.), *p* < 0.001, *d* = 1.69). Descriptive data are shown in Table 2.

## Discussion

The present study aimed to investigate the influence of cuff stiffness of elastic cuffs on OTO, v_sys_, muscle oxygenation, and perceived cuff pressure in young males and females. In general, the results indicate that cuff stiffness affected blood flow velocity and perceived cuff pressure, whereas muscle oxygenation was not influenced. In particular, when the cuff was stiffer (i) arterial occlusion was achieved with less %overlap and (ii) with progressive overlap, the decrease in v_sys_ and the increase in perceived cuff pressure were higher. Furthermore, (iii) the decrease in S_m_O_2_ and increase in tHb did not differ between cuffs but (iv) sexes, with lower S_m_O_2_ and higher tHb in males compared to females.

The cuff with the highest stiffness (i.e., HS cuff) required the smallest %overlap to occlude the arterial blood flow, as indicated by the lowest OTO compared to the two other cuffs. This was expected, given that the HS cuff compresses the underlying tissue and vessels to a larger extent at a given overlap compared to a cuff with a lower stiffness. This result is supported by the v_sys_ data. It was shown that with increasing overlap the decline in v_sys_ was steeper when the cuff stiffness was higher, indicating that a stiffer cuff leads to a greater reduction in blood flow velocity compared to a cuff with less stiffness, when an equal %overlap is applied. In a comparable approach, Mouser et al. [13] also assessed v_sys_ in the brachial artery, however, by using pneumatic cuffs with different cuff widths (5, 10, and 12 cm). The authors observed a distinct drop in v_sys_ of 29.7% compared to rest, when applying a 5 cm wide cuff with a cuff pressure corresponding to 80% AOP [13]. To transfer these results to the present study, an overlap of 30% or 20% using the MS cuff (-30.4%) or HS cuff (-28.5%), respectively, might have produced a similar cuff pressure of about 80% AOP. Nevertheless, this transfer remains speculative. In general, the cuff pressure selected for BFR or pBFR should be high enough to strongly reduce or occlude the venous blood flow but lower than the AOP or OTO, respectively, to avoid arterial blood flow occlusion. Abe et al. [11] already stated that the force-elongation curve of commercial elastic cuffs for pBFR (i.e., elastic knee wrap (Harbinger Red-Line, Fairfield, CA, USA, 7.6 cm width)) exceed linearity, because cuffs with a low stiffness may enter the plastic-elastic region (i.e. force-elongation curve is flattening) before an adequate pBFR pressure has been generated. In this regard, arterial occlusion was not possible in three participants with an arm circumference of ≥ 37 cm when using the cuffs with lower stiffness (MS and LS cuff), indicating that stiffer cuffs may be more suitable for people with large arm circumference. Furthermore, by using stiffer cuffs, the OTO might be within the linear zone of the force-elongation relationship. Consequently, it seems possible to set a specific percentage OTO to create an individualized pressure, which corresponds to the current recommendations for a safe application of BFR at rest and during exercise (i.e., 40–80% AOP [10]). However, this should be investigated in further studies to make more precise recommendations.

Contrary to our hypothesis, S_m_O_2_ and tHb decreased and increased with progressive overlap, respectively, but were not affected by the cuff stiffness, meaning that the greater reduction in v_sys_ generated by the HS cuff did not contribute to a higher change in muscle oxygenation or venous blood pooling. These findings are in contrast to the results by Cunniffe et al. [23], who have found a greater reduction in S_m_O_2_ with increasing pressure during 3 cycles of 5 min at rest with three different cuff pressures (140, 160, and 180 mmHg). However, the differences in S_m_O_2_ in the upper limbs were only between the 140 mmHg and the two higher pressures. The authors hypothesized that pressures > 140 mmHg might occlude blood flow in the upper limbs, which could explain the similar changes in S_m_O_2_ in the 160 and 180 mmHg pressure conditions [23]. Nevertheless, even the subocclusive pressures (10% and 20% overlap) did not induce different changes in S_m_O_2_ between the three cuffs in the present study. An explanation for this finding might be the restriction time which was 30 s per %overlap and therefore resulted in a total restriction time of 1:30 min. Hence, it might be assumed that the restriction time used in the present study was too short to induce differences in muscle oxygenation between the elastic cuffs. In this regard, McLay et al. [24] showed that longer durations of vascular occlusion (i.e., 1, 2, 3, and 5 min) lead to a greater decline in S_m_O_2_ compared to 30 s. Therefore, it can be assumed that longer restriction times might have led to differences in S_m_O_2_ and tHb between the cuffs. Nevertheless, sex differences were found with lower S_m_O_2_ and higher tHb in males compared to females. This might be due to a greater proportional area of type I muscle fibers [11] and lower hemoglobin levels in females [25].

From a practical point of view, the application using a %overlap is a simple method to standardize the pBFR pressure between exercises and training sessions [4]. However, this technique is hardly reproducible, if the material properties are unknown (e.g., using a different elastic cuff [11]). Nevertheless, the present study also revealed that the perceived cuff pressure was highest when the HS cuff was applied, although the %overlap was the same compared to the other two cuffs. Considering this result, the cuff application based on the perceived cuff pressure might lead to comparable amounts of pBFR pressure regardless of the cuff stiffness and therefore, seems suitable for the application when the user is not aware of the material properties [4]. However, the RPP scale value at OTO was 7.0 ± 1.5, 7.3 ± 1.5, and 8.1 ± 1.1 out of 10 for the HS, MS, and LS cuff, respectively. Therefore, using the RPP scale by Wilson et al. [5] with the target of 7 on a scale up to 10 (i.e., moderate pressure with no pain), which is commonly used for pBFR exercise, might lead to an underestimation of the applied pressure and might be occlusive in some cases. Furthermore, the perceived pressure technique might also be only valid for the initial application because it is less reliable over time [26]. Even between exercises, the application based on the perceived cuff pressure might be biased, because exercise-induced hypoalgesia might lead to the application of higher pBFR pressures. This assumption is based on the results of studies that have shown a higher pressure pain thresholds, indicating lower pressure/pain perception, immediately and 5 min after 4 sets of an isometric handgrip exercise at 30% 1RM with 50% AOP [27] as well as 5 min and 24 h following 4 sets of unilateral leg press exercise at 30% 1RM with 40% and 80% AOP [28]. Therefore, it is important to have an individually tailored pBFR pressure, which is reliable between exercises and training sessions (i.e., relative overlap technique) [4].

A first limitation of the present study is that the influence of cuff stiffness was examined at rest, which might limit the relevance for practitioners. Therefore, future research is needed to investigate the influence of cuff stiffness on acute and chronic adaptations in response to pBFR combined with exercise. Furthermore, future studies should also record the blood flow volume when comparing cuffs with different stiffness, given that changes in blood flow volume seem to be different compared to those in v_sys_ [13]. Moreover, the results of the present study are limited to the upper limbs, which does not allow to draw conclusions about the influence of the cuff stiffness on hemodynamics and perceived cuff pressure in the lower extremities. These aspects should also be investigated in future studies.

## Conclusions

The present study showed that cuff stiffness influences v_sys_ and must be considered for pBFR like other already known influencing factors such as arm circumference and cuff width [13, 29]. However, the perceived cuff pressure was also affected by cuff stiffness and, therefore, setting the cuff pressure based on participants’ pressure perception seems feasible for the initial application, when the material properties are unknown [4]. Nevertheless, the RPP scale [5], which is commonly used for pBFR exercise, might underestimate the applied pressure when targeting a 7 on a scale up to 10. Furthermore, it appears that stiffer cuffs are more suitable for pBFR compared to cuffs with less stiffness, given that they are more effective in restricting the blood flow. By recording the %overlap at arterial occlusion, the practitioner can set a pressure in relation to the OTO (given that the cuff stiffness is linear up to this point). Moreover, stiffer cuffs might be needed for people with larger arm circumferences (e.g., weight lifters, bodybuilders), because cuffs with less stiffness seem to generate an insufficient pressure for the restriction of blood flow. However, future research should investigate whether the influence of cuff stiffness on the dependent variables is only valid for subjects who are at rest (i.e., passive BFR) or also plays a role when pBFR is combined with exercise/training.

## Data Availability

The datasets used and analyzed during the current study are available from the corresponding author on reasonable request.

## Abbreviations

1RM: One repetition maximum
AOP: Arterial occlusion pressure
BFR: Blood flow restriction
HS: High stiffness
LS: Low stiffness
MS: Medium stiffness
OTO: Overlap to occlusion
pBFR: Practical blood flow restriction
RPP: Rating of perceived cuff pressure
v_sys_: Systolic peak blood flow velocity
a.u.: Arbitary unit
